# Substance of Lectures on Gold

**Published:** 1878-05

**Authors:** 


### ARTICLE VI.

### *Substance of Lectures on Gold.*

BY PROFESSOR HODGKIN.

We have spent sufficient time over the details of mining to have become somewhat familiar with the processes employed, and this evening are prepared to proceed to what to us, is a most important division—*the refining* of the metal, and its separation from its metallic associations. We will bear in mind the fact already mentioned, that in dealing with gold we do not meet with natural combinations—any chlorides or oxides of gold we may have to deal with are formed artificially by us by the aid of chemicals, and are not to be met with in a natural form. But we saw what to the metallurgist is of great importance, that certain other metals, some of them noble, and others not belonging to that class, were associated with the gold, and were alloyed with it, and as it is of the utmost importance to us that the gold we deal with should be *pure*, it is a grave question with us how we shall obtain that purity without which the yellow metal is useless. To illustrate how exceedingly important it is that the baser metals, if any, should be thoroughly removed, it is stated that two grains of lead, or one half a grain of antimony will render one thousand grains of gold unmalleable. No work therefore is more exact, and no processes in metallurgy must be conducted with greater nicety than that of refining gold ; so that if we spend some little time over it you will understand that it is because of the precision required. But lest some should say beforehand that it is too complex a study for students not far advanced in this branch of research, I will state that there is but one quality necessary on your part—that of close attention. A great man has said that the main difference between minds is that of attention,—the ability to give perfect, full attention to a subject, constituting in his judgmen the disparity between the weak and the strong. If this be true, and I am much inclined to believe in the doctrine, it is only necessary for us to give our minds fully to this or any other subject in order that we may thoroughly understand it. And certainly in this branch of chemistry we will find it plain sailing, if we fix a few cardinal facts in our minds, and study a few simple chemical laws, and their action.

The books on metallurgy make much use of a term which at first seemed to us may be puzzling—the *discrimination* of gold. It means, in my plain definition, the processes for deciding, (1) what is gold, and, (2) how much gold is in a certain mass of alloy, or ore.

And first as to what is gold. This is a most interesting question, as mistakes have been and are frequently made by persons not experts in metallurgy, such errors sometimes being of a grave character and involving immense loss, and occasionally of a ludicrous nature. Several different mineral substances have somewhat the lustre and appearance of the precious metal. *Mica* in small scales glistens as we see it scattered among the sand of the highway, and its glitter is sometimes mistaken for gold. A magnifying glass will easily enable one to discern the difference—the peculiar translucent scales of the mica showing plainly under an ordinary hand-glass. *Iron Pyrites*, (ferrous sulphide) containing one atom of iron and two of sulphur, has still more frequently been mistaken for gold. Its peculiar crystallization will enable us to discriminate this mineral and place it in its proper class. The blow-pipe, that invaluable and indispensable companion to the metallurgist, will readily reduce the iron, which gives off sulphur vapors, and by the application of a magnet we have a ready and certain test of its nature. It is worth while, in passing, to say that this iron pyrites, although a nearly valueless ore, so far as its iron is concerned, is of considerable value for another purpose in the arts, as from it is made most of the ferrous sulphate or copperas of commerce, as well as sulphuric acid, and also a considerable amount of sulphur and alum.

As these are the minerals we are most likely to mistake for gold, I will leave the qualitatives and pass on to the quantitative discrimination, and only pause to say that in the office of the assayer the nicest tests are required and made use of in this most interesting branch of metallurgy, as it is of the greatest importance to know whether it will pay to separate the gold from the mass of other materials with which it is mingled, or to extract it from the metals with which it is alloyed; in other words to determine the amount of gold present in the mass we are to examine. The assaying of gold ores does not concern us specially, but it is interesting to be able to estimate the amount of gold in alloys, as most of that which we are likely to handle is of this character.

The old method of the "touch-stone" was a fair approximate test of the value of gold alloys, and though only approximate was much depended on before the more modern and exact processes now employed. To practice this method it was necessary to have a sample of gold of known value, and several grades were kept for this purpose, drawn into large wire, or hammered into pieces called "needles." These ranged from fine gold by half carats down to eighteen, or lower still if desired. Thus the first would be 24c, the second 23½c, &c.

"The touch-stone itself is simply a hard piece of basaltic rock, with a surface sufficiently hard to abrade the metal and retain upon its surface enough of the piece to be tested for examination by acid." Nitric acid is used for this purpose. The test-bars are rubbed on the stone, leaving their mark, and the alloy to be tested is similarly treated. If the action of the acid upon the piece of *unknown* quality is the same as upon that which is *known*, we have an appoximate test of the value of the specimen we are experimenting upon. The acid develops a green color on coming in contact with the alloying metals, while the gold is of course untouched. This method will be seen to be rude and uncertain, yet it is useful in some cases, as it has the merit of being easily applied, and is sufficiently accurate for much of the testing we would require. Much depends upon the judgment. It will be observed also that it leaves us without knowledge of the character of the alloying metals, a point of great importance, as we will see further on.

Far more accurate methods, involving great precision of manipulation and exact results are described in the works on metallurgy, but they involve costly and, to us nearly useless apparatus ; and the methods are almost too complex and require too accurate a knowledge of the finer arts, to say nothing of the science of assaying, to be of practical use to us. But a very simple method for determining the amount of gold in a given case would be to take say one part of the alloy to be tested for gold, and two or three parts of silver and about seven of lead. This is "cupelled," as it is termed. (The word and the method are very old, and the process one of great beauty and simplicity. A cupel is literally a "little cup," and in this case is a crucible made of bone-ash, dampened and stamped into a steel mould. The operation is founded on the well-known property of characterising the precious metals, viz., that when heated to fusion and exposed to a current of air they do not oxydize, while the base metals associated with them and undergoing similar treatment do oxydize. By this simple means we are enabled to be rid of the impure metals. The unoxydizable metals require different treatment.) On applying the blast of the blow-pipe to our little assay the whole mass is fused, and the lead, carrying with it any other oxydizable metals which may be present, is absorbed into the cupel. The gold and silver are parted by Aqua Regia, and the gold precipitated and weighed.

Of the gold which falls into the hands of the dental mechanician, only that which is in the form of foil is pure, and even some foils are slightly alloyed to destroy or lessen their cohesiveness. Gold coins are alloyed definitely as to quantity, and with some at least definitely as to quality. The gold plate for sale in the depots should be of known valuation, and probably is in many cases ; while the old gold plates which come to us as dentists for repair, or are purchased as old material, are of course unknown, unless made by ourselves. Of these it may be said that the solder contains always considerable copper and silver, and occasionally possibly zinc, while the platinum pins in the backings further complicate the question of refining the mass.

It is not however impossible, nor even greatly difficult for us not only to comprehend the processes by which gold may be refined, but if we will give the subject a little careful study, and our manipulation of the several processes that intelligent care which as dentists we should bring to bear on all the studies we engage in, and all the work we perform, we will find that the difficulties of this subject, which classes are usually shy of, will vanish, and I shall get as intelligent answers from you as students on the subject of "refining gold" as on the familiar one of Vulcanite.

[TO BE CONTINUED.]

